# Perceived Discrimination in Health Care Settings and Care Delays in Patients With Diabetes and Hypertension

**DOI:** 10.1001/jamanetworkopen.2025.0046

**Published:** 2025-03-04

**Authors:** Maryam Jafari Bidgoli, Hui Wang, Casey Macander, Abbey Gregg

**Affiliations:** 1 Department of Community Medicine and Population Health, College of Community Health Sciences, The University of Alabama, Tuscaloosa; 2 Institute for Rural Health Research, The University of Alabama, Tuscaloosa

## Abstract

**Importance:**

Some US populations experience perceived discrimination in health care settings (PDHS) that may lead to delaying health care and worse health outcomes. It is crucial to understand potential mechanisms, such as patient-clinician communication (PCC), that may reduce these disparities.

**Objective:**

To examine PCC as a mediating pathway between PDHS and health care delays due to nervousness about seeing a health care professional while considering the moderating roles of age and race and ethnicity.

**Design, Setting, and Participants:**

This cross-sectional study examined data from the All of Us Research Program between May 31, 2017, and April 30, 2022. Included participants were adults aged 18 years or older with type 2 diabetes and/or hypertension. The data analysis was performed between February 20 and April 29, 2024.

**Exposure:**

Perceived discrimination in the health care setting.

**Main Outcome and Measures:**

The study outcome was health care delays due to being nervous about seeing a health care professional. The association between PDHS and health care delays was examined through PCC using a structural equation model with moderated mediation analysis.

**Results:**

The analytic sample comprised 25 581 participants (mean [SD] age, 62.4 [12.8] years; 58.1% women; and 9.5% of Black, 6.3% of Hispanic, 81.3% of White, and 2.9% of other race and ethnicity). Delaying health care due to feeling nervous about seeing a health care professional was reported by 2300 participants (8.9%). Patient-clinician communication mediated the association between PDHS and delayed care. Mediation proportions decreased with age across all racial and ethnic groups, with the highest proportions observed among participants aged 18 to 44 years (Black participants, 19.5% [95% CI, 14.9%-24.1%]; Hispanic participants, 20.3% [95% CI, 15.6%-25.1%]; White participants, 19.0% [95% CI, 14.7%-23.2%]; participants of other race and ethnicity, 18.3% [95% CI, 13.2%-23.5%]). Within each age group, the highest mediation proportions were observed among Hispanic participants, followed by Black and White participants, and participants of other race and ethnicity.

**Conclusions and Relevance:**

These findings suggest that higher PDHS is positively associated with delaying health care due to nervousness about seeing a health care professional. The largest mediation proportion observed was among younger adults and racial and ethnic minority groups. By prioritizing better PCC, health care delays associated with patient apprehension related to perceived discrimination may be reduced.

## Key Points

**Question:**

What is the association of patient-clinician communication (PCC) with perceived discrimination in health care settings (PDHS) and health care delays due to nervousness about seeing a health care professional among US adults with type 2 diabetes and/or hypertension?

**Findings:**

In this cross-sectional study of 25 851 participants in the All of Us Research Program, perceived discrimination in health care settings was positively associated with delaying health care due to nervousness about seeing a health care professional. Poor PCC linked to these delays partially mediated the association, with mediation proportions varying by race and ethnicity and age.

**Meaning:**

These findings suggest that improved PCC may reduce health care delays associated with patient apprehension related to perceived discrimination.

## Teaser

This cross-sectional study examines whether patient-clinician communication is associated with perceived discrimination in health care settings and care delays among US patients with chronic conditions.

## Introduction

Within the health care setting, patients should feel assured of comfort and safety. However, up to 20% of Americans report discrimination during medical interactions.^1^ Perceived discrimination in health care settings (PDHS), defined as experiences of being treated unfairly based on such characteristics as race, ethnicity, and age, may harm both mental and physical health.^2^ These experiences decrease trust in health care professionals; disrupt communication; discourage health care–seeking behavior^3,4,5^; and are associated with delayed care, poor treatment adherence, underuse of preventive services, and reduced quality of care.^6,7,8,9,10,11,12^

Despite considerable knowledge about the effect of discrimination on health care use, the underlying mechanisms remain understudied, and delays associated with nervousness^13^ about seeing a health care professional have been largely overlooked. Experiencing discrimination may trigger a heightened stress response,^14^ and in this context, nervousness about visiting a health care professional may be linked to prior negative experiences, such as PDHS. These negative experiences may further discourage individuals from seeking timely care.

Poor communication may exacerbate the negative effects of discrimination,^15^ further hindering access to timely and appropriate care. Miscommunication, misunderstanding, and bias may have negative consequences for diagnosis, treatment decisions, and patient satisfaction.^16^ Patient-clinician communication (PCC) plays a crucial role in shaping health behaviors, outcomes, and use.^17^ Strong PCC promotes positive behaviors and outcomes among patients with chronic diseases.^18,19,20^

Perceptions of discrimination and its association with communication may vary based on the reason for discrimination, such as age or race and ethnicity.^21,22^ Ageist stereotypes, such as assumptions about reduced autonomy or cognitive decline, may contribute to older adults communicating less assertively and participating less in decision-making. These biases may also result in clinicians dismissing these patients’ concerns, worsening the quality of care and patient-clinician interactions.^23,24^ Similarly, racial and ethnic minority patients may alter their communication styles because of past discrimination, affecting PCC.

Patients with chronic conditions such as type 2 diabetes and hypertension need regular visits for effective disease management. These common diseases, which are leading modifiable risk factors for premature death, are often poorly managed, with racial and ethnic disparities in outcomes.^25,26,27^ Investigating the role of PCC in the association between PDHS and delays in care is vital for addressing systemic inequities and advancing health care equity.^28^

To fill this gap, we leveraged a large, geographically diverse sample of patients with type 2 diabetes and/or hypertension from the All of Us Research Program (AoU) to examine whether PCC mediates the association between PDHS and delayed health care due to nervousness. We hypothesized the following: (1) the likelihood of increased health care delays due to feeling nervous about seeing a health care professional may be associated with increasing levels of PDHS, (2) PCC may partially mediate the association between PDHS and delays in seeing a health care professional due to nervousness, and (3) age and race and ethnicity may moderate the proportion of the association mediated through PCC.

## Methods

In this cross-sectional study, data from AoU participants were collected via online surveys between May 31, 2017, and April 30, 2022. The aim of the AoU is to gather health-related information from more than 1 million people in the US for a wide coverage of geographic areas.^29^ The AoU was approved by the National Institutes of Health Institutional Review Board. Individuals’ deidentified data were approved to be shared with investigators through the AoU Research Hub. The University of Alabama Institutional Review Board considered this study exempt from review because only deidentified data were accessed through the Registered Tier of the AoU Research Hub. All AoU participants provided informed consent to participate in the study, with the protocol approved and actively monitored by the AoU Institutional Review Board. This study followed the Strengthening the Reporting of Observational Studies in Epidemiology (STROBE) reporting guideline for cross-sectional studies.^30^

### Study Population

Participants in the AoU were invited to complete many surveys covering a wide range of topics. Once participants finished the core surveys (The Basics, Lifestyle, and Overall Health), they proceeded to additional topic-specific surveys. Our study included all adults aged 18 years or older with type 2 diabetes and/or hypertension who participated in both the Health Care Access and Utilization (HCAU) and Social Determinants of Health surveys.^31^ Participants with incomplete demographic information were excluded. Diabetes and hypertension diagnoses came from review of historical electronic health record data shared with the AoU, which resulted in a cohort of 25 851 participants with at least 1 diagnostic record of diabetes or hypertension disorder (Systematized Nomenclature of Medicine codes 73211009 and 38341003, respectively) before their registration date with AoU (eFigure 1 in Supplement 1).

### Outcome

The primary outcome measure was health care delay due to feeling nervous about seeing a health care professional, which was derived from responses to the following question in the HCAU survey: “Have you delayed seeking medical care in the past 12 months due to feeling nervous about seeing a health care provider?” First, participants were asked about how long it had been since they last saw or talked to a physician or other health care professional about their own health. If the response was more than 1 year, they were asked to select from a list of reasons why many people delay getting medical care (eTable 1 in Supplement 1). Possible reasons included lack of transportation, distance to the health care professional, inability to get time off work, lack of childcare, and other reasons of which one was feeling nervous about seeing a health care professional (eTable 2 in Supplement 1). From this point on, we refer to health care delays due to nervousness simply as health care delays.

### Exposure

The 7-item PDHS (Cronbach α = 0.90) is an adapted scale from the Everyday Discrimination Scale, which assesses the participant’s prior treatment experiences while receiving health care services. Responses are measured on a 5-point Likert scale.^32,33^ Respondents rate the frequency of experiencing discrimination from never to always, with a score of 1 to 5 assigned for each level of response. A summary of responses to each question is available in eTables 3 and 4 in Supplement 1. A total of 438 individuals who answered 4 or fewer questions on the 7-item scale were excluded from the analysis to ensure reliability. A continuous discrimination score was created by taking the mean score of responses to this question set. A higher score represents experiencing more discrimination in medical settings.

### Covariates

The AoU collects demographic information from participants through The Basics core survey^31^ during registration. Self-reported race and ethnicity categories were regrouped to address model convergence challenges. We categorized race and ethnicity into Black (non-Hispanic Black or African American), Hispanic (Hispanic Asian; Hispanic Black or African American; Hispanic Middle Eastern or North African; Hispanic more than 1 population; Hispanic Native Hawaiian or Pacific Islander; Hispanic, race not indicated; and Hispanic White), White (non-Hispanic White), and Other (non-Hispanic Asian, non-Hispanic Middle Eastern or North African, non-Hispanic Native Hawaiian or Pacific Islander, and non-Hispanic more than 1 population) (eTable 5 in Supplement 1). Age groups were classified as 18 to 44 years, 45 to 64 years, and 65 years or older. The models were adjusted for potential confounding factors, including self-reported gender (man; woman; and nonbinary, transgender, or other [combined per AoU policy regarding the reporting of small sample sizes]), marital status (married or partnered; never married; divorced, separated, or widowed), education (no college, college, advanced degree), income ($0-$24 999, $25 000-$49 999, $50 000-$74 999, $75 000-$99 999, ≥$100 000), and sexual orientation (heterosexual, not heterosexual).

### Mediator

Patient-clinician communication was measured using 2 questions from the HCAU survey that asked participants about how often their physician or health care professional (1) asked for their opinions or beliefs about their medical care or treatment (eg, what kind of tests, procedures, or medications they prefer) and (2) told or gave them information about their health and health care that was easy to understand (eTables 6 and 7 in Supplement 1). These questions assess key aspects of PCC. The first question evaluates shared decision-making, reflecting how health care professionals involve patients in decisions about tests, treatments, or medications, which enhances satisfaction, care quality, and clinician communication.^34^ The second question focuses on clarity of communication, assessing how well health care professionals convey information to support patient comprehension and informed decision-making, reducing patient uncertainty and nervousness.^35^ Responses ranged from always to none of the time, with scores of 1 to 4 assigned to each level. The mean combined score was used to represent the overall level of PCC. Higher PCC scores indicated poorer communication.

### Statistical Analysis

A structural equation model is a statistical framework for simultaneous model regression equations and tests the hypothetical associations among many variables.^36^ We used a structural equation model with moderated mediation analysis.^37^ We included PCC as a potential mediator of the association between PDHS (exposure) and health care delays (outcome). Moderated mediation analysis with 2 moderators was performed to understand the proportion of the mediated pathway in the association between PDHS and health care delays and the roles of race and ethnicity and age as moderators of this mechanism. Then, we estimated mediation proportions for different values of one moderator when the second moderator was held fixed. This study used data from the AoU’s Controlled Tier Dataset, version 7, available to authorized users on the Research Workbench.^38^ All statistical analyses were performed between February 20 and April 29, 2024, in the AoU Research Workbench (a cloud-based platform) using R, version 4.3.1 (R Foundation). False discovery rate adjustment was applied to the *P* values to control for multiple comparisons and determine statistical significance, which was set at a 2-sided *P* < .05.

**Figure 1.**
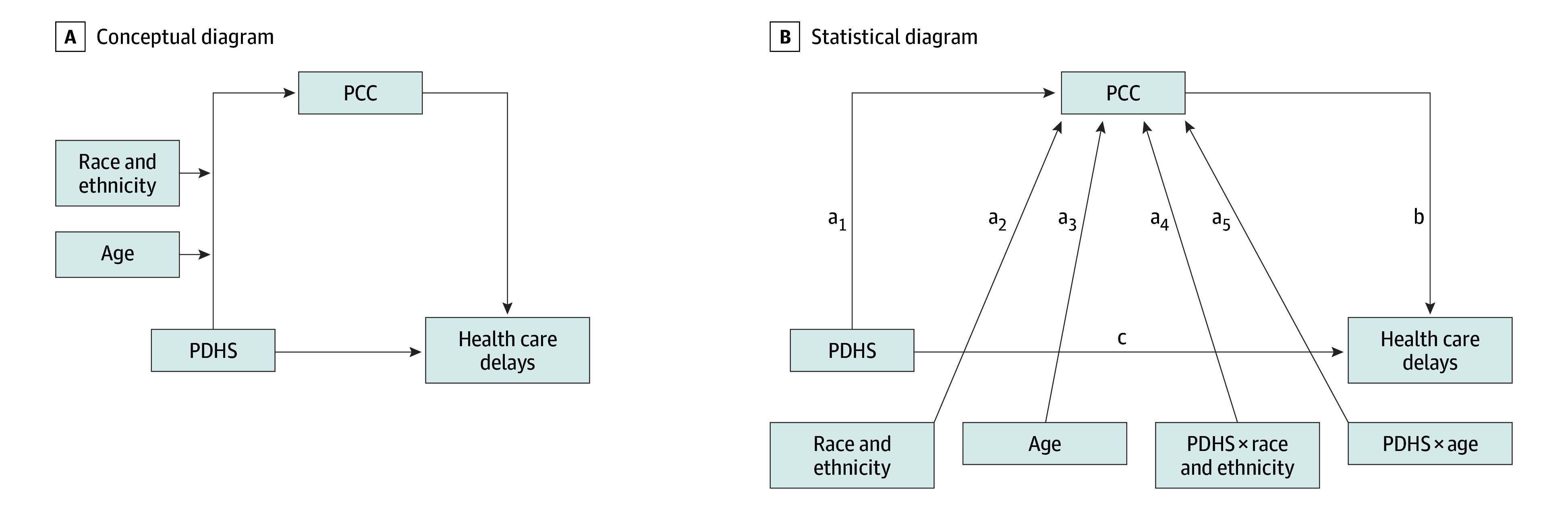
Moderated Mediation Analysis of Associations Among Perceived Discrimination in Health Care Settings (PDHS), Patient-Clinician Communication (PCC), and Health Care Delays A, Hypothesized associations, with PDHS as the exposure variable, PCC serving as the mediator, and race and ethnicity and age acting as moderators of the mediated pathway. B, Coefficient estimates (a_1_-a_5_, b, and c) quantify the associations among PDHS (exposure variable), PCC (mediator), and health care delays (outcome), with race and ethnicity and age moderating the mediated pathway. Health care delay is defined as delays due to feeling nervous about seeing a health care professional. For simplicity, other covariates included in the model are not shown.

Figure 1 shows the study’s conceptual and statistical frameworks. The pathway from PDHS to PCC is moderated by age and race and ethnicity under the assumption that one moderator is fixed to be independent of the other. The association between PDHS and PCC depends on both age and race and ethnicity and equals a_1_ + a_4_ (race and ethnicity) + a_5_ (age), and the association between PCC and health care delays is represented by b. Therefore, the mediated association of PDHS with health care delays through PCC is measured by a_1_ b + a_4_ b (race and ethnicity) + a_5_ b (age).

## Results

Our sample included 25 851 individuals with type 2 diabetes and/or hypertension documented in their electronic health record who also participated in the HCAU and Social Determinants of Health surveys. The sample population had a mean (SD) age of 62.4 (12.8) years, and 41.3% identified as men; 58.1% as women; and 0.5% as nonbinary, transgender, or other gender. Most of the sample self-reported White race (81.3% compared with 9.5% who reported Black, 6.3% Hispanic, and 2.9% other race and ethnicity). Most participants reported being in at least good health (36.7% in good health, 34.1% in very good health, and 7.4% in excellent health). Of our total sample, 2300 (8.9%) reported delaying health care due to being nervous about seeing a health care professional (Table 1; eTable 8 in Supplement 1).

**Table 1.** Sample Characteristics (N = 25 851)

Characteristic	Participants, No. (%)		
	Health care delay^a^		Total (N = 25 851)^b^
	Yes (n = 2300 [8.9%])	No (n = 21 943 [84.9%])	
Age, y			
18-44	646 (28.1)	2052 (9.4)	2770 (10.7)
45-64	1098 (47.7)	8258 (37.6)	9859 (38.1)
≥65	556 (24.1)	11 633 (53.0)	13 222 (51.2)
Gender			
Man	611 (26.6)	9445 (43.0)	10 684 (41.3)
Woman	1655 (72.0)	12 397 (56.5)	15 028 (58.1)
Nonbinary, transgender, other	34 (1.5)	101 (0.5)	139 (0.5)
Sexual orientation			
Heterosexual	1988 (86.4)	20 446 (93.2)	23 941 (92.6)
Not heterosexual	312 (13.6)	1497 (6.8)	1910 (7.4)
Race and ethnicity			
Black	213 (9.3)	2057 (9.4)	2444 (9.5)
Hispanic	192 (8.4)	1334 (6.1)	1638 (6.3)
White	1817 (79.0)	17 922 (81.7)	21 013 (81.3)
Other^c^	78 (3.4)	630 (2.9)	756 (2.9)
Education			
No college	303 (13.1)	2627 (12.0)	3178 (12.3)
College	1417 (61.6)	12 034 (54.8)	14 349 (55.5)
Advanced degree	580 (25.2)	7282 (33.2)	8324 (32.2)
Marital status			
Married or partnered	1264 (55.0)	13 996 (63.8)	16 180 (62.6)
Never married	455 (19.8)	2540 (11.9)	3160 (12.2)
Divorced, separated, or widowed	581 (25.3)	5407 (24.6)	6511 (25.2)
Income, $			
0-24 999	568 (24.7)	3176 (14.5)	4088 (15.8)
25 000-49 999	486 (21.1)	3906 (17.8)	4737 (18.3)
50 000-74 999	350 (15.2)	3841 (17.5)	4510 (17.5)
75 000-99 999	283 (12.3)	3192 (14.6)	3676 (14.2)
≥100 000	613 (26.7)	7828 (35.7)	8840 (34.2)
Self-reported health rating			
Poor	210 (9.1)	664 (3.0)	927 (3.6)
Fair	684 (29.7)	3619 (16.5)	4563 (17.7)
Good	888 (38.6)	8025 (36.6)	9474 (36.7)
Very good	444 (19.3)	7789 (35.5)	8824 (34.1)
Excellent	60 (2.6)	1730 (7.9)	1913 (7.4)
Skip	14 (0.6)	116 (0.5)	150 (0.6)
PDHS score, mean (SD)^d^	1.92 (0.72)	1.50 (0.60)	1.54 (0.62)
PCC score, mean (SD)^e^	2.11 (0.71)	1.78 (0.66)	1.81 (0.67)

Abbreviations: PCC, patient-clinician communication; PDHS, perceived discrimination in health care setting.

^a^ Health care delays due to feeling nervous about seeing a health care professional.

^b^ Health care delay skip, no applicable, do not know (n = 1608) are included in the total.

^c^ Other includes non-Hispanic Asian, non-Hispanic Middle Eastern or North African, non-Hispanic Native Hawaiian or Pacific Islander, and non-Hispanic more than 1 population.

^d^ A higher score indicates greater perceived discrimination in the health care setting (maximum score, 5.00).

^e^ A higher score indicates poorer PCC (maximum score, 4.00).

The structural equation model was fit using the robust maximum likelihood method. The structural model fit the data well (eResults in Supplement 1). Table 2 presents coefficient estimates for PCC and health care delays, 95% CIs, and *P* values for the variables depicted in Figure 1B.

**Table 2.** Coefficient Estimates for PCC and Health Care Delays^a^

Variable^c^	Outcome					
	PCC			Health care delays^b^		
	Label^d^	β (95% CI)	*P* value	Label^d^	β (95% CI)	*P* value
Mediator						
PCC^e^	NA	NA	NA	b	0.25 (0.21 to 0.28)	<.001
Exposure						
PDHS^f^	a_1_	0.33 (0.30 to 0.36)	<.001	c	0.35 (0.27 to 0.43)	<.001
Race and ethnicity						
Black	a_2_	−0.13 (−0.20 to −0.06)	<.001	NA^g^	−0.23 (−0.45 to −0.01)	.04
Hispanic	a_2_	−0.07 (−0.15 to 0.01)	.07	NA^g^	−0.02 (−0.27 to 0.23)	.88
Other^h^	a_2_	0.02 (−0.10 to 0.14)	.77	NA^g^	−0.30 (−0.71 to 0.11)	.16
Age, y						
45-64	a_3_	0.05 (−0.02 to 0.12)	.14	NA^g^	−0.21 (−0.39 to −0.03)	.02
≥65	a_3_	0.14 (0.07 to 0.21)	<.001	NA^g^	−0.68 (−0.87 to −0.49)	<.001
PDHS × race and ethnicity						
Black	a_4_	0.01 (−0.02 to 0.05)	.50	NA	NA	NA
Hispanic	a_4_	0.03 (−0.01 to 0.07)	.17	NA	NA	NA
Other^h^	a_4_	−0.01 (−0.08 to 0.05)	.69	NA	NA	NA
PDHS × age						
45-64 y	a_5_	−0.05 (−0.09 to −0.02)	.003	NA	NA	NA
≥65 y	a_5_	−0.12 (−0.15 to −0.08)	<.001	NA	NA	NA
Gender						
Man	NA	NA	NA	NA^g^	−0.21 (−0.27 to −0.16)	<.001
Nonbinary, transgender, or other	NA	NA	NA	NA^g^	0.07 (−0.18 to 0.32)	.58
Education						
College	NA^g^	0.02 (−0.01 to 0.04)	.19	NA^g^	0.11 (0.03 to 0.19)	.006
Advanced degree	NA^g^	−0.02 (−0.05 to 0.02)	.31	NA^g^	0.07 (−0.02 to 0.16)	.12
Income, $						
25 000-49 999	NA^g^	0.02 (−0.01 to 0.05)	.11	NA^g^	−0.05 (−0.13 to 0.03)	.19
50 000-74 999	NA^g^	0.02 (−0.01 to 0.06)	.12	NA^g^	−0.18 (−0.26 to −0.09)	<.001
75 000-99 999	NA^g^	0.01 (−0.03 to 0.04)	.62	NA^g^	−0.20 (−0.29 to −0.11)	<.001
≥100 000	NA^g^	0.00 (−0.03 to 0.03)	.82	NA^g^	−0.24 (−0.32 to −0.15)	<.001
Sexual orientation						
Not heterosexual	NA	NA	NA	NA^g^	0.17 (0.08 to 0.25)	<.001
Marital status						
Married or partnered	NA	NA	NA	NA^g^	−0.07 (−0.14 to 0.01)	.07
Divorced, separated, or widowed	NA	NA	NA	NA^g^	−0.06 (−0.14 to 0.02)	.14

Abbreviations: NA, not applicable; PCC, patient-clinician communication; PDHS, perceived discrimination in health care setting.

^a^ Health care delays due to feeling nervous about seeing a health care professional.

^b^ The coefficient estimates for the health care delays equation indicate the changes in standardized *z* scores, given in units of change in the estimator.

^c^ Reference groups were White, woman, no college, income $0 to $24 999, heterosexual, unmarried, and aged 18 to 44 years.

^d^ Labels are shown in Figure 1B.

^e^ A higher score indicates poorer PCC.

^f^ A higher score indicates greater PDHS.

^g^ For simplicity, the coefficient label for the covariate is not shown in Figure 1B.

^h^ Other includes non-Hispanic Asian, non-Hispanic Middle Eastern or North African, non-Hispanic Native Hawaiian or Pacific Islander, and non-Hispanic more than 1 population.

As shown in Table 2, higher PDHS levels were significantly associated with increased health care delays due to nervousness (c: β = 0.35; 95% CI, 0.27-0.43; *P* < .001). Participants with higher scores in PCC (indicating poorer communication) were significantly more likely to report health care delays due to nervousness (b: β = 0.25; 95% CI, 0.21-0.28; *P* < .001). A significant, positive association was also observed between PDHS and PCC (a_1_: β = 0.33; 95% CI, 0.30-0.36; *P* < .001) (eFigure 2 in Supplement 1).

Table 3 presents the estimated coefficient for the direct pathway (PDHS to health care delay) and the calculated coefficients for the mediated pathway (PDHS to PCC to health care delay), along with the mediation proportions. The extent to which the association between PDHS and health care delays was mediated by PCC varied across demographic groups. Among Hispanic participants, the mediation proportions were largest across all age groups (18-44 years: 20.3% [95% CI, 15.6%-25.1%]; 45-64 years: 17.9% [95% CI, 13.3%-22.5%]; ≥65 years: 14.7% [95% CI, 10.5%-18.9%]) compared with the other racial and ethnic groups. Black participants exhibited similar trends, with the mediation proportions decreasing with age (18-44 years: 19.5% [95% CI, 14.9%-24.1%]; 45-64 years: 17.0% [95% CI, 12.7%-21.3%]; ≥65 years: 13.8% [95% CI, 9.8%-17.7%]). Mediation proportions also decreased with age among White participants (18-44 years: 19.0% [95% CI, 14.7%-23.2%]; 45-64 years: 16.4% [95% CI, 12.5%-20.4%]; ≥65 years: 13.2% [95% CI, 9.8%-16.5%]) and for participants of other race and ethnicity (18-44 years: 18.3% [95% CI, 13.2%-23.5%]; 45-64 years: 15.8% [95% CI, 10.8%-20.8%]; ≥65 years: 12.4% [95% CI, 7.6%-17.3%]).

**Table 3.** Calculated Coefficient of Pathways in the Association Between PDHS and Health Care Delays^a^ and Mediation Proportions

Pathway	β (95% CI)	*P* value
Direct (PDHS to health care delay)	0.351 (0.273-0.428)	<.001
Mediated pathway (PDHS to PCC to health care delay)		
Black participants		
Aged 18-44 y	0.085 (0.069-0.101)	<.001
Aged 45-64 y	0.072 (0.059-0.085)	<.001
Aged ≥65 y	0.056 (0.044-0.068)	<.001
Hispanic participants		
Aged 18-44 y	0.089 (0.072-0.106)	<.001
Aged 45-64 y	0.076 (0.061-0.091)	<.001
Aged ≥65 y	0.061 (0.047-0.074)	<.001
White participants		
Aged 18-44 y	0.082 (0.072-0.106)	<.001
Aged 45-64 y	0.069 (0.061-0.091)	<.001
Aged ≥65 y	0.053 (0.047-0.074)	<.001
Participants of other race and ethnicity^b^		
Aged 18-44 y	0.079 (0.059-0.099)	<.001
Aged 45-64 y	0.065 (0.047-0.084)	<.001
Aged ≥65 y	0.050 (0.032-0.068)	<.001
Mediation proportion, %^c^		
Black participants		
Aged 18-44 y	19.5 (14.9-24.1)	<.001
Aged 45-64 y	17.0 (12.7-21.3)	<.001
Aged ≥65 y	13.8 (9.8-17.7)	<.001
Hispanic participants		
Aged 18-44 y	20.3 (15.6-25.1)	<.001
Aged 45-64 y	17.9 (13.3-22.5)	<.001
Aged ≥65 y	14.7 (10.5-18.9)	<.001
White participants		
Aged 18-44 y	19.0 (14.7-23.2)	<.001
Aged 45-64 y	16.4 (12.5-20.4)	<.001
Aged ≥65 y	13.2 (9.8-16.5)	<.001
Participants of other race and ethnicity^b^		
Aged 18-44 y	18.3 (13.2-23.5)	<.001
Aged 45-64 y	15.8 (10.8-20.8)	<.001
Aged ≥65 y	12.4 (7.6-17.3)	<.001

Abbreviations: PCC, patient-clinician communication; PDHS, perceived discrimination in health care setting.

^a^ Health care delays due to feeling nervous about seeing a health care professional.

^b^ Other includes non-Hispanic Asian, non-Hispanic Middle Eastern or North African, non-Hispanic Native Hawaiian or Pacific Islander, and non-Hispanic more than 1 population.

^c^ Proportion of the total association (direct + mediated pathway) between PDHS and health care delays mediated by PCC.

**Figure 2.**
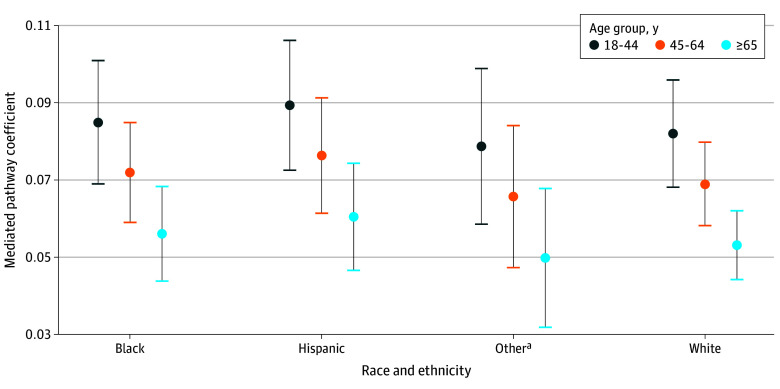
Mediated Pathway Coefficients for the Indirect Association Between Perceived Discrimination in Health Care Settings (PDHS) and Health Care Delays Through Patient-Clinician Communication (PCC) Coefficients are a function of age and race and ethnicity. The mediated pathway (indirect association between PDHS and health care through PCC) is measured as a_1 _b + a_4 _b (race and ethnicity) + a_5 _b (age). Error bars represent the 95% CIs for the coefficients. ^a^Other includes non-Hispanic Asian, non-Hispanic Middle Eastern or North African, non-Hispanic Native Hawaiian or Pacific Islander, and non-Hispanic more than 1 population.

Figure 2 displays the size of the mediated pathway in the association between PDHS and health care delays through PCC as a function of age group and race and ethnicity. Across all groups, the decline in the mediation proportion with age suggests that PCC’s mediating role diminished in older populations while maintaining variability across racial and ethnic groups.

## Discussion

In this cross-sectional study of 25 851 participants with type 2 diabetes and/or hypertension in the AoU, 8.9% reported delays in seeking medical care due to nervousness about seeing a health care professional. We found significant associations between PDHS and PCC and health care delays. In addition, PDHS was significantly associated with PCC. Finally, PCC mediated the association between PDHS and delays in health care due to nervousness, with mediation proportions varying by age and race and ethnicity. Mediation proportions decreased with age across all racial and ethnic groups, with the highest proportions observed among individuals aged 18 to 44 years. Within each age group (18-44, 45-64, ≥65 years), the highest mediation proportions were observed in Hispanic individuals (20.3%, 17.9%, and 14.7%, respectively), followed by Black individuals (19.5%, 17.0%, 13.8%, respectively), White individuals (19.0%, 16.4%, and 13.2%, respectively), and individuals of other races and ethnicities (18.3%, 15.8%, and 12.4%, respectively).

Our findings add to previous studies that have shown that negative experiences with health care professionals are associated with patients’ decisions to seek treatment.^39^ Higher PDHS may lead to delayed necessary care^6,10,11^ and may influence health outcomes.^40^ Such delays may result from mistrust related to discriminatory experiences,^41^ as patients with PDHS often experience negative emotions, which have been observed to be a major barrier to attending appointments.^42^ Importantly, our study builds upon existing studies by showing that communication, specifically shared medical decision-making and clarity of information, may explain the association between discrimination and health care delays due to feeling nervous about visiting a health care professional. This mediation effect was stronger for younger adult and racial and ethnic minority patients. Improving communication between patients and health care professionals may be a key pathway to reducing delays in health care due to nervousness, particularly for patients who perceive discrimination.

Among patients aged 18 to 44 years, a larger portion of the delays in care associated with discrimination was explained by PCC compared with older patients, regardless of race and ethnicity. Age plays an important role in the patient-clinician interaction^43^ and may shape how patients perceive communication with health care professionals. This difference may be influenced by several factors, including generational shifts in health care dynamics. Older adults, who experienced health care during a time when traditional, hierarchical dynamics dominated,^44^ may not view ineffective communication as an important barrier to care.^45^ They may have less of a desire for involvement in medical decision-making,^46^ reflecting a history of deference to physicians and limited emphasis on patient-centered care.^47^ Ageism may also influence health care communication, as physicians may involve older patients in decision-making less frequently than they involve younger patients,^23^ further diminishing the perceived importance of effective communication for this group.

Younger patients, on the other hand, may prioritize autonomy, value shared decision-making, and be more sensitive to communication quality,^45^ all of which align with modern health care models emphasizing patient-centered care and collaboration. Clinicians may engage younger patients with more interactive communication styles that meet the patients’ expectations, making communication a more crucial factor in health care delays. Addressing ageist biases in health care communication is essential to ensure that older adults are actively engaged in their care and to minimize disparities in health care outcomes across age groups.

Racial and ethnic differences in mediation proportions, while relatively small, highlight the unique challenges faced by minority populations. Hispanic participants consistently showed the highest mediation proportions across all age groups, followed by Black participants, White participants, and participants of other races and ethnicities. These findings align with broader evidence that perceptions of disrespect or unfair treatment in patient-clinician interactions are prevalent, especially among racial and ethnic minority patients.^10^ Patients who have experienced discrimination may expect future negative encounters, which might harm communication with health care professionals.^15^ Perceived discrimination also fosters medical mistrust,^48^ reducing disclosure and adherence to medical advice. Among racial and ethnic minority patients, difficulty understanding physicians has been linked to perceived discrimination, further worsening communication and health care experiences.^49^

A diverse health care workforce that represents the growing racial and ethnic minority population in the US is fundamental to improving the patient experience. Building this workforce requires educational pipeline programs that lead to health professional schools, supporting the ongoing development and retention of a diverse workforce and providing training on cultural competency and communication skills.^50^ Communication and interpersonal skills building courses are associated with improved physician communication, patient satisfaction, and patient experience.^51^ Cultural competence training for health care professionals is associated with improved clinician knowledge and skills, with some improvements in patient satisfaction; however, more rigorous research with validated assessments of cultural competence, patient satisfaction, discrimination, and other objective measures are still needed.^52,53,54^ Future studies of clinician communication and antidiscrimination interventions should consider incorporating patient health care delays as a potential objective measure to assess the outcomes associated with such interventions.

### Limitations

This study has several limitations. The cross-sectional design restricted our ability to infer causal relationships, and the self-reported survey data introduced a potential for response bias or social desirability bias. While the AoU has been purposefully recruiting participants from historically underrepresented populations, it relies on convenience sampling, making our findings not representative of the general population. Ascertainment bias is possible as electronic health record data are currently available only for participants associated with a health care professional organization funded by AoU. Our study’s focus on patients with type 2 diabetes and/or hypertension limited the generalizability of findings to other conditions. Finally, while the study controlled for many sociodemographic factors, unmeasured confounding variables such as health care navigation, health literacy, individual behaviors, and language proficiency may still have influenced the results.

## Conclusions

In this cross-sectional study of individuals with type 2 diabetes and/or hypertension, PDHS was associated with health care delays due to nervousness about visiting a health care professional. Poor PCC mediated this association, with the extent of mediation varying across age and racial and ethnic groups. Addressing discrimination in the health care system is essential to encouraging timely health care–seeking behaviors, particularly for patients requiring regular care. Improving PCC is equally crucial to reducing health care delays that may stem from nervousness.

## Notes

Supplement 1. **eFigure 1.** Flow Diagram of the Sample Selection **eTable 1.** Time Since Last Contact With a Health Care Professional **eTable 2.** Proportion of Health Care Delays Attributed to Specific Reasons Among Respondents **eTable 3.** The 7-Item Perceived Discrimination in Health Care Setting Response Rate **eTable 4.** Summary of Responses to the 7-Item Perceived Discrimination in Health Care Settings **eTable 5.** Race and Ethnicity Reclassification **eTable 6.** The 2-Item Patient-Clinician Communication Response Rate **eTable 7.** Summary of Responses to the 2-Item Patient-Clinician Communication
eResults.
**eTable 8.** Sample Characteristics by Perceived Discrimination in Health Care Setting and Patient-Clinician Communication (n = 25 851) **eFigure 2.** Association Between Perceived Discrimination in Health Care Settings (PDHS) and Patient-Clinician Communication (PCC) by Race and Ethnicity and Age
eReferences

Supplement 2.
Data Supplement
