# Supplementary material for: Perceived Discrimination in Health Care Settings and Care Delays in Patients With Diabetes and Hypertension
Source: JAMA Netw Open. 2025 Mar 4;8(3):e250046. doi: 10.1001/jamanetworkopen.2025.0046 (PMC11880957; doi:10.1001/jamanetworkopen.2025.0046)
Supplement: Supplement 1. — eFigure 1. Flow Diagram of the Sample Selection eTable 1. Time Since Last Contact With a Health Care Professional eTable 2. Proportion of Health Care Delays Attributed to Specific Reasons Among Respondents eTable 3. The 7-Item Perceived Discrimination in Health Care Setting Response Rate eTable 4. Summary of Responses to the 7-Item Perceived Discrimination in Health Care Settings eTable 5. Race and Ethnicity Reclassification eTable 6. The 2-Item Patient-Clinician Communication Response Rate eTable 7. Summary of Responses to the 2-Item Patient-Clinician Communication eResults. eTable 8. Sample Characteristics by Perceived Discrimination in Health Care Setting and Patient-Clinician Communication (n = 25 851) eFigure 2. Association Between Perceived Discrimination in Health Care Settings (PDHS) and Patient-Clinician Communication (PCC) by Race and Ethnicity and Age eReferences [file jamanetwopen-e250046-s001.pdf]

Supplemental Online Content

Jafari Bidgoli M, Wang H, Macander C, Gregg A. Perceived discrimination in health care settings and care delays in patients with diabetes and hypertension. *JAMA Netw Open*. 2025;8(3):e250046. doi:10.1001/jamanetworkopen.2025.0046

eFigure 1. Flow Diagram of the Sample Selection

eTable 1. Time Since Last Contact With a Health Care Professional

eTable 2. Proportion of Health Care Delays Attributed to Specific Reasons Among Respondents

eTable 3. The 7-Item Perceived Discrimination in Health Care Setting Response Rate

eTable 4. Summary of Responses to the 7-Item Perceived Discrimination in Health Care Settings

eTable 5. Race and Ethnicity Reclassification

eTable 6. The 2-Item Patient-Clinician Communication Response Rate

eTable 7. Summary of Responses to the 2-Item Patient-Clinician Communication

eResults

eTable 8. Sample Characteristics by Perceived Discrimination in Health Care Setting and Patient-Clinician Communication (n = 25 851)

eFigure 2. Association Between Perceived Discrimination in Health Care Settings (PDHS) and Patient-Clinician Communication (PCC) by Race and Ethnicity and Age

eReferences

This supplemental material has been provided by the authors to give readers additional information about their work.

© 2025 Jafari Bidgoli M et al. *JAMA Network Open*.

Methods

Study Population

eFigure 1. Flow Diagram of the Sample Selection

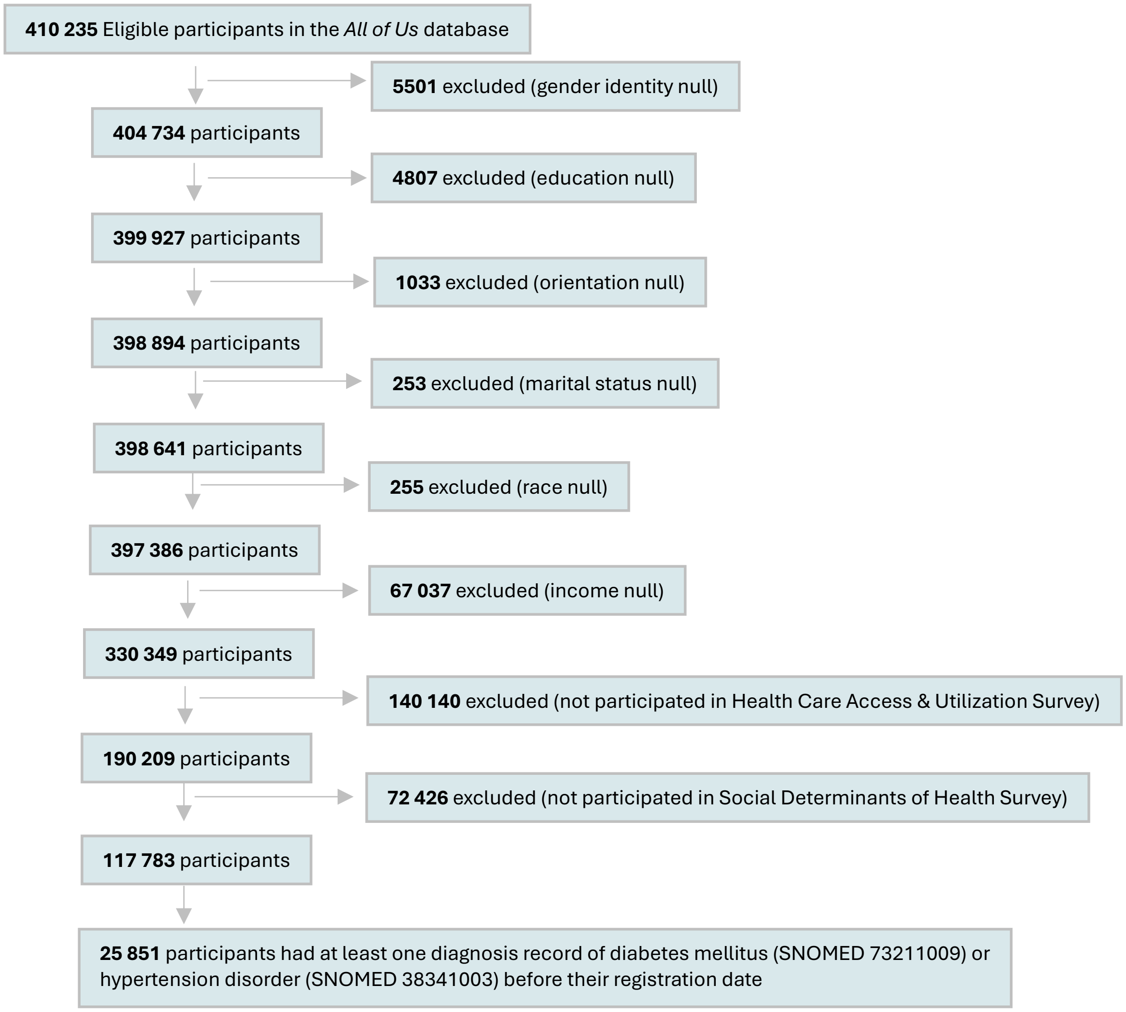

© 2025 Jafari Bidgoli M et al. *JAMA Network Open*.

Outcome

From Health Care Access and Utilization Survey (HCAU) "*About how long has it been since you last saw or talked to a doctor or other health care provider about your own health?*"

eTable 1. Time Since Last Contact With a Health Care Professional

Response	No. (%)
1. Never	68 (0.26)
2. 6 months or less	23 470 (90.79)
3. More than 6 months, but not more than 1 year ago	1723 (6.67)
4. More than 1 year, but not more than 2 years ago	267 (1.03)
5. More than 2 years, but not more than 5 years ago	49 (0.19)
6. More than 5 years ago	17 (0.07)
7. Don't know	257 (0.99)

If selected 4, 5 or 6, skip to the question "*There are many reasons people delay getting medical care. Have you delayed getting care for any of the following reasons in the PAST 12 MONTHS?*"

eTable 2. Proportion of Health Care Delays Attributed to Specific Reasons Among Respondents

Reason	No. (%)
1. Didn't have transportation	1514 (5.86)
2. You live in a rural area where distance to the health care provider is too far.	669 (2.59)
3. You were nervous about seeing a health care provider.	2300 (8.90)
4. Couldn't get time off work.	1399 (5.41)
5. Couldn't get child care.	361(1.40)
6. You provide care to an adult and could not leave him/her.	425 (1.64)
7. Couldn't afford the copay.	1476 (5.71)
8. Your deductible was too high/or could not afford the deductible.	1638 (6.34)
9. You had to pay out of pocket for some or all of the procedure.	3027(11.71)

© 2025 Jafari Bidgoli M et al. *JAMA Network Open*.

Exposure

Assessment of Perceived Discrimination in Health Care Settings (PDHS)

How often do any of these happen to you when you go to a doctor's office or other health care provider?

(Never, Rarely, Sometimes, Most of the time, Always)

1. You are treated with less courtesy than other people.
2. You are treated with less respect than other people.
3. You receive poorer service than others.
4. A doctor or nurse acts if he or she thinks you are not smart.
5. A doctor or nurse acts as if he or she is afraid of you.
6. A doctor or nurse acts as if he or she is better than you.
7. You feel like a doctor or nurse is not listening to what you were saying.

eTable 3. The 7-Item Perceived Discrimination in Health Care Setting Response Rate

Item	No. (%)[a]
1. You are treated with less courtesy than other people.	25 503 (98.66)
2. You are treated with less respect than other people.	25 330 (97.99)
3. You receive poorer service than others.	25 179 (97.40)
4. A doctor or nurse acts if he or she thinks you are not smart.	25 000 (96.70)
5. A doctor or nurse acts as if he or she is afraid of you.	24 129 (93.34)
6. A doctor or nurse acts as if he or she is better than you.	25 377 (98.17)
7. You feel like a doctor or nurse is not listening to what you were saying.	25 305 (97.88)

[a] The percentages represent the response rates for each question, which were assessed using a 5-point Likert scale.

eTable 4. Summary of Responses to the 7-Item Perceived Discrimination in Health Care Settings

Item	No. (%)
1. You are treated with less courtesy than other people.	
Always	345 (1.33)
Most of the time	227 (0.88)
Never	14 962 (57.88)
Skip	334 (1.29)
Rarely	7506 (29.04)
Sometimes	2463 (9.53)
NA	14 (0.05)
2. You are treated with less respect than other people.	
Always	300 (1.16)
Most of the time	211 (0.82)
Never	14 921 (57.72)
Skip	494 (1.91)
Rarely	7379 (28.54)
Sometimes	2519 (9.74)
NA	27 (0.10)
3. You feel like a doctor or nurse is not listening to what you were saying.	
Always	315 (1.22)
Most of the time	808 (3.13)
Never	10 592 (40.97)
Skip	521 (2.02)
Rarely	8103 (31.35)
Sometimes	5487 (21.23)
NA	25 (0.10)

© 2025 Jafari Bidgoli M et al. *JAMA Network Open.*

4. You receive poorer service than others.	
Always	288 (1.11)
Most of the time	212 (0.82)
Never	15 211 (58.84)
Skip	645 (2.50)
Rarely	7254 (28.06)
Sometimes	2214 (8.56)
NA	27 (0.10)
5. A doctor or nurse acts as if he or she is afraid of you.	
Always	252 (0.97)
Most of the time	52 (0.20)
Never	21 746 (84.12)
Skip	1663 (6.43)
Rarely	1730 (6.69)
Sometimes	349 (1.35)
NA	59 (0.23)
6. A doctor or nurse acts as if he or she is better than you.	
Always	194 (0.75)
Most of the time	461 (1.78)
Never	15 229 (58.91)
Skip	458 (1.77)
Rarely	6541 (25.30)
Sometimes	2952 (11.42)
NA	16 (0.06)
7. A doctor or nurse acts if he or she thinks you are not smart.	
Always	318 (1.23)
Most of the time	337 (1.30)
Never	16 640 (64.37)
Skip	816 (3.16)
Rarely	5387 (20.84)
Sometimes	2318 (8.97)
NA	35 (0.14)

© 2025 Jafari Bidgoli M et al. *JAMA Network Open*.

Covariates

eTable 5. Race and Ethnicity Reclassification

All of Us **Research Program**		**n**	**Our sample**
Ethnicity	Race		
Hispanic or Latino	Asian	7	collapsed into Hispanic 1638
Hispanic or Latino	Black or African American	30	
Hispanic or Latino	Middle Eastern or North African	4	
Hispanic or Latino	More than 1 population	30	
Hispanic or Latino	Native Hawaiian or Pacific Islander	1	
Hispanic or Latino	None Indicated	1292	
Hispanic or Latino	White	274	
Not Hispanic or Latino	Asian	355	collapsed into Other 756
Not Hispanic or Latino	Middle Eastern or North African	97	
Not Hispanic or Latino	Native Hawaiian or Pacific Islander	13	
Not Hispanic or Latino	More than 1 population	291	
Not Hispanic or Latino	Black or African American	2444	
Not Hispanic or Latino	White	21 013	

© 2025 Jafari Bidgoli M et al. *JAMA Network Open*.

Mediator

Assessment of Communication between Patient and Health Care Clinician (PCC)

1. How often did your doctors or health care providers ask for your opinions or beliefs about your medical care or treatment? For example, what kind of tests, procedures, or medications you prefer. Would you say…
2. How often did your doctors or health care providers tell or give you information about your health and health care that was easy to understand? Would you say…

(Always, Most of the time, Some of the time, None of the time, Don't know)

eTable 6. The 2-Item Patient-Clinician Communication Response Rate

Item	No. (%)
1. How often did your doctors or health care providers ask for your opinions or beliefs about your medical care or treatment? For example, what kind of tests, procedures, or medications you prefer. Would you say…	24 919 (96.39)
2. How often did your doctors or health care providers tell or give you information about your health and health care that was easy to understand? Would you say…	25 130 (97.21)

eTable 7. Summary of Responses to the 2-Item Patient-Clinician Communication

Item	No. (%)
1. How often did your doctors or health care providers ask for your opinions or beliefs about your medical care or treatment?	
Always	7286 (28.18)
Don't know	322 (1.25)
Most of the time	8249 (31.91)
None of the time	2895 (11.20)
Skip	234 (0.91)
Some of the time	6489 (25.10)
NA	376 (1.45)
2. How often did your doctors or health care providers tell or give you information about your health and health care that was easy to understand?	
Always	15 831 (61.24)
Don't Know	48 (0.19)
Most Of The Time	8152 (31.53)
None Of The Time	109 (0.42)
Skip	297 (1.15)
Some Of The Time	1038 (4.02)
NA	376 (1.45)

© 2025 Jafari Bidgoli M et al. *JAMA Network Open*.

eResults

Model Fitness

Structural equation model (SEM) was fitted using the robust maximum likelihood method, which is known to perform well in SEM with categorical variables. The goodness of fit was assessed using Chi-Square (χ^2), Root Mean Square Error of Approximation (RMSEA), Comparative Fit Index (CFI), Tucker-Lewis Index (TLI), and Standardized Root Mean Square Residual (SRMR).[1] The structural model fit the data well ($\chi^2(10) = 11.97, RMSEA$=.0029, CFI=.9898, TLI=.9989 and $SRMR$=.0001).[2]

© 2025 Jafari Bidgoli M et al. *JAMA Network Open*.

eTable 8. Sample Characteristics by Perceived Discrimination in Health Care Setting and Patient-Clinician Communication (n = 25 851)

Characteristic	PDHS[a] No. (SD)	PCC[b] No. (SD)
Age, y		
18-44	1.77 (0.73)	1.91 (0.73)
45-64	1.62 (0.66)	1.83 (0.69)
≥65	1.44 (0.55)	1.78 (0.64)
Gender		
Man	1.46 (0.60)	1.77 (0.66)
Woman	1.60 (0.63)	1.83 (0.68)
Nonbinary, transgender, or other	1.94 (0.75)	1.93 (0.77)
Sexual orientation		
Heterosexual	1.53 (0.61)	1.81 (0.67)
Not heterosexual	1.72 (0.72)	1.86 (0.70)
Race and ethnicity		
Black	1.70 (0.73)	1.76 (0.73)
Hispanic	1.56 (0.65)	1.82 (0.75)
White	1.52 (0.60)	1.81 (0.66)
Other[c]	1.64 (0.68)	1.84 (0.68)
Education		
No college	1.56 (0.69)	1.80 (0.75)
College	1.57 (0.63)	1.83 (0.68)
Advanced degree	1.49 (0.58)	1.78 (0.63)
Marital status		
Married or partnered	1.50 (0.59)	1.80 (0.65)
Never married	1.70 (0.71)	1.85 (0.71)
Divorced, separated, or widowed	1.58 (0.64)	1.82 (0.70)
Income, $		
0-24 999	1.72 (0.73)	1.84 (0.76)
25 000-49 999	1.60 (0.65)	1.83 (0.70)
50 000-74 999	1.52 (0.59)	1.82 (0.66)
75 000-99 999	1.51 (0.59)	1.80 (0.64)
≥100 000	1.46 (0.56)	1.78 (0.64)
Self-reported health rating		
Poor	1.92 (0.78)	2.02 (0.77)
Fair	1.72 (0.70)	1.93 (0.73)
Good	1.56 (0.61)	1.83 (0.66)
Very good	1.44 (0.55)	1.74 (0.63)
Excellent	1.35 (0.54)	1.66 (0.64)
Skip	1.52 (0.68)	1.74 (0.71)

Abbreviations: PCC, patient-clinician communication; PDHS, perceived discrimination in health care setting.
[a] A higher score indicates greater perceived discrimination in health care setting (maximum score, 5.00).
[b] A higher score indicates poorer patient-clinician communication (maximum score, 4.00).
[c] Other includes non-Hispanic Asian, non-Hispanic Middle Eastern or North African, non-Hispanic Native Hawaiian or Pacific Islander and non-Hispanic more than 1 population.

© 2025 Jafari Bidgoli M et al. *JAMA Network Open*.

eFigure 2. Association Between Perceived Discrimination in Health Care Settings (PDHS) and Patient-Clinician Communication (PCC) by Race and Ethnicity and Age

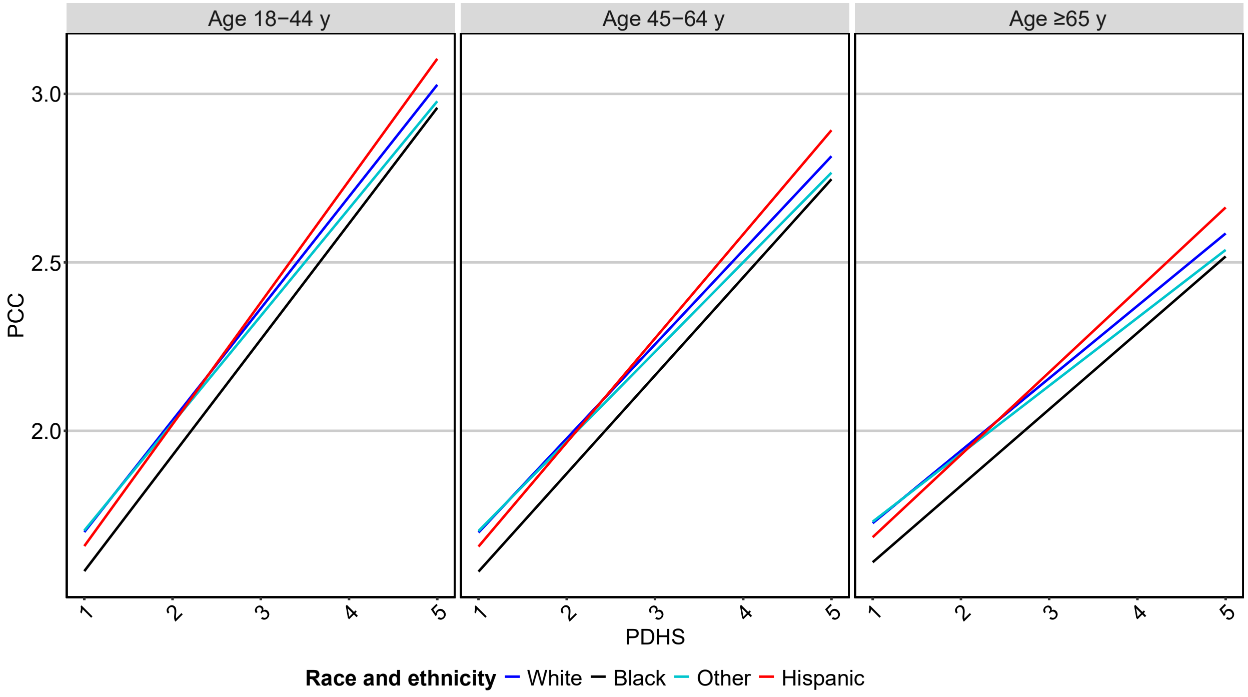

The lines depict the association between PDHS and PCC, with the slopes calculated as: $a_1 + a_4$ (race and ethnicity) + a_5 (age), where a_1, a_4 and a_5 are the estimated coefficients from Table 2. The slopes vary by race and ethnicity and age, ranging from 0.32 to 0.36 for ages 18–44, decreasing to 0.27–0.31 for ages 45–64, and further declining to 0.20–0.24 for ages 65 and older.

© 2025 Jafari Bidgoli M et al. *JAMA Network Open*.

eReferences

1. Hooper D, Coughlan J, Mullen MR. Structural equation modelling: Guidelines for determining model fit. *Electronic Journal of Business Research Methods*. 2008;6(1):53-60.
2. Satorra A, Bentler PM. Model conditions for asymptotic robustness in the analysis of linear relations. *Computational Statistics & Data Analysis*. 1990;10(3):235-249. doi:10.1016/0167-9473(90)90004-2

© 2025 Jafari Bidgoli M et al. *JAMA Network Open*.
