# Supplementary material for: Perceived Discrimination in Health Care Settings and Care Delays in Patients With Diabetes and Hypertension
Source: JAMA Netw Open. 2025 Mar 4;8(3):e250046. doi: 10.1001/jamanetworkopen.2025.0046 (PMC11880957; doi:10.1001/jamanetworkopen.2025.0046)
Supplement: Supplement 2. — Data Supplement [file jamanetwopen-e250046-s002.pdf]

Data Sharing Statement

Jafari Bidgoli. Perceived Discrimination in Health Care Settings and Care Delays in Patients With Diabetes and Hypertension. *JAMA Netw Open*. Published March 04, 2025.
doi:10.1001/jamanetworkopen.2025.0046

Data
Data available: No

Additional Information
Explanation for why data not available: The data used in this research were obtained from the "All of Us" Research Program, which requires that all statistical analyses be conducted within the All of Us Research Workbench, a secure, cloud-based platform. As such, data cannot be downloaded or shared externally in compliance with the program's data use policies.
